# Higher mortality and intubation rate in COVID-19 patients treated with noninvasive ventilation compared with high-flow oxygen or CPAP

**DOI:** 10.1038/s41598-022-10475-7

**Published:** 2022-04-20

**Authors:** Sergi Marti, Anne-Elie Carsin, Júlia Sampol, Mercedes Pallero, Irene Aldas, Toni Marin, Manel Lujan, Cristina Lalmolda, Gladis Sabater, Marc Bonnin-Vilaplana, Patricia Peñacoba, Juana Martinez-Llorens, Julia Tárrega, Óscar Bernadich, Ana Córdoba-Izquierdo, Lourdes Lozano, Susana Mendez, Eduardo Vélez-Segovia, Elena Prina, Saioa Eizaguirre, Ana Balañá-Corberó, Jaume Ferrer, Judith Garcia-Aymerich

**Affiliations:** 1 Respiratory Department. Hospital, Universitari Vall d’Hebron, Passeig Vall d’Hebron, 119-129, 08035 Barcelona, Spain; 2 Universitat Autònoma de Barcelona (UAB), Barcelona, Spain; 3 CIBER de Enfermedades Respiratorias (CIBERES), Madrid, Spain; 4 ISGlobal, Barcelona, Spain; 5 Universitat Pompeu Fabra (UPF), Barcelona, Spain; 6 CIBER Epidemiología y Salud Pública (CIBERESP), Madrid, Spain; 7 Respiratory Department, Hospital Universitari Germans Trias i Pujol, Badalona, Spain; 8 Respiratory Department, Corporació Sanitària Parc Tauli, Sabadell, Spain; 9 Department of Pulmonology, Dr. Josep Trueta, University Hospital of Girona, Santa Caterina Hospital of Salt, Girona, Spain; 10 Girona Biomedical Research Institute (IDIBGI), Girona, Spain; 11 Respiratory Department, Hospital de la Santa Creu i Sant Pau, Barcelona, Spain; 12 Respiratory Department, Hospital del Mar, Barcelona, Spain; 13 Respiratory Department, Hospital General de Granollers, Granollers, Spain; 14 Universitat Internacional de Catalunya, Barcelona, Spain; 15 Respiratory Department, Althaia Xarxa Assistencial Universitària de Manresa, Manresa, Spain; 16 Respiratory Department, Hospital Universitari de Bellvitge, L’Hospitalet de Llobregat, Llobregat, Spain; 17 Respiratory Department, Hospital Mútua de Terrassa, Terrassa, Spain

**Keywords:** Respiratory distress syndrome, Infectious diseases

## Abstract

The effectiveness of noninvasive respiratory support in severe COVID-19 patients is still controversial. We aimed to compare the outcome of patients with COVID-19 pneumonia and hypoxemic respiratory failure treated with high-flow oxygen administered via nasal cannula (HFNC), continuous positive airway pressure (CPAP) or noninvasive ventilation (NIV), initiated outside the intensive care unit (ICU) in 10 university hospitals in Catalonia, Spain. We recruited 367 consecutive patients aged ≥ 18 years who were treated with HFNC (155, 42.2%), CPAP (133, 36.2%) or NIV (79, 21.5%). The main outcome was intubation or death at 28 days after respiratory support initiation. After adjusting for relevant covariates and taking patients treated with HFNC as reference, treatment with NIV showed a higher risk of intubation or death (hazard ratio 2.01; 95% confidence interval 1.32–3.08), while treatment with CPAP did not show differences (0.97; 0.63–1.50). In the context of the pandemic and outside the intensive care unit setting, noninvasive ventilation for the treatment of moderate to severe hypoxemic acute respiratory failure secondary to COVID-19 resulted in higher mortality or intubation rate at 28 days than high-flow oxygen or CPAP. This finding may help physicians to choose the best noninvasive respiratory support treatment in these patients.

Clinicaltrials.gov identifier: NCT04668196.

## Introduction

The spread of the pandemic caused by the coronavirus SARS-CoV-2 has placed health care systems around the world under enormous pressure. Up to 10–15% of hospitalized cases with coronavirus disease 2019 (COVID-19) are in critical condition (i.e., severe pneumonia and hypoxemic acute respiratory failure, HARF), have received invasive mechanical ventilation, and are admitted to the intensive care unit (ICU)^1,2^. The shortage of critical care resources, both in terms of equipment and trained personnel, required a reorganization of the hospital facilities even in developed countries. In addition, some COVID-19 patients cannot be considered for invasive ventilation due to their frailty or comorbidities, and others are unwilling to undergo invasive techniques. As a result, a considerable proportion of severe patients are being treated in hospital settings outside the ICU.

Noninvasive respiratory support (NIRS) techniques, including high-flow oxygen administered via nasal cannula (HFNC), continuous positive airway pressure (CPAP) and noninvasive ventilation (NIV), have been used in severe COVID-19 patients, although their use was initially controversial due to doubts about its effectiveness^3–6^, and the risk of aerosol-linked infection spread^7^. Initial recommendations^8–12^ were based on previous evidence in non-COVID patients and early experience during the pandemic, but they differed in terms of the type of NIRS proposed as first option, and lacked COVID-specific evidence to support them.

So far, observational COVID-19 studies have suggested that either HFNC, CPAP or NIV may improve oxygenation and reduce the need for intubation or the risk of death^13–18^, but the effects of different NIRS techniques have been compared in few studies^16,19,20^. An observational study analyzing 670 patients found no differences in 30-day mortality or endotracheal intubation between HFNC, CPAP and NIV used outside the ICU, after adjusting for confounders^16^. In contrast, a randomized study of 110 COVID-19 patients admitted to the ICU found no differences in the 28-day respiratory support-free days (primary outcome) or mortality between helmet NIV and HFNC, but recorded a lower risk of endotracheal intubation with helmet NIV (30%, vs. 51% for HFNC)^19^.

Overall, the information supporting the choice of one or other NIRS technique is limited. Moreover, the COVID-19 pandemic is still active around the world, and data supporting an evidence-based choice of NIRS are urgently needed. In this multicentre, observational real-life study, we aimed to compare the effects of high-flow oxygen administered via nasal cannula, continuous positive airway pressure, and noninvasive ventilation, initiated outside the intensive care unit, in preventing death or endotracheal intubation at 28 days in patients with COVID-19.

## Methods

### Study design

A multicentre, retrospective cohort study of COVID-19 patients followed from NIRS initiation up to 28 days or death, whichever occurred first.

### Study population

**Figure 1:**
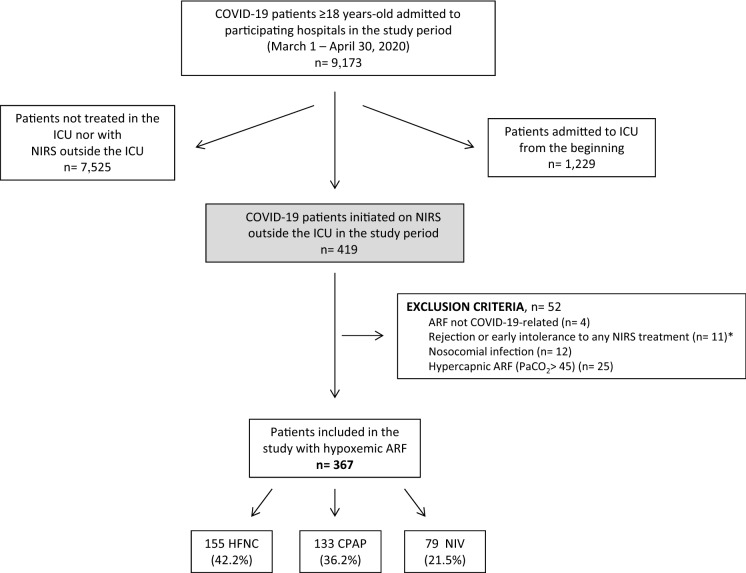
Flowchart. *ARF* acute respiratory failure, *HFNC* high-flow nasal cannula, *ICU* intensive care unit, *NIRS* non-invasive respiratory support, *NIV* non-invasive ventilation. *HFNC, n = 2; CPAP, n = 6; NIV, n = 3. In addition, 26 patients who presented early intolerance were treated subsequently with other NIRS treatment, and were included as study patients in this second treatment: 8 patients with intolerance to HFNC (2 patients treated subsequently with CPAP, and 6 with NIV), 11 patients with intolerance to CPAP (5 treated later with HFNC, and 6 with NIV), and 7 patients with intolerance to NIV (5 treated after with HFNC, and 2 with CPAP).

We included a consecutive sample of patients aged at least 18 years who had initiated NIRS treatment for HARF related to COVID-19 pneumonia outside the ICU at any of the 10 participating university hospitals, during the first pandemic surge, between 1 March and 30 April 2020. All participating hospitals belong to the National Health System of Catalonia, Spain, and attend a population of around 4.3 million inhabitants. COVID-19 diagnosis was confirmed through reverse-transcriptase-polymerase-chain-reaction assays performed on nasopharyngeal swab specimens. From a total of 419 candidate patients, we excluded those with: (1) respiratory failure not related to COVID-19 (e.g., cardiogenic pulmonary edema as primary cause of respiratory failure); (2) rejection or early intolerance to any NIRS treatment; (3) pregnancy; (4) nosocomial infection; and (5) PaCO_2_ above 45 mm Hg. A total of 367 patients were finally included in the study (Fig. 1), which was approved by the research ethics committee at each participating hospital (study coordinator centre, Hospital Vall d'Hebron, Barcelona; protocol No. PR(AG)265/2020). Research was performed in accordance with the Declaration of Helsinki. The requirement of informed consent was waived due to the retrospective nature of the study.

### Treatment strategies

The NIRS treatments evaluated were high-flow oxygen administered via nasal cannula (HFNC), continuous positive airway pressure (CPAP), and noninvasive ventilation (NIV). According to current Spanish recommendations^8^, criteria for initiating respiratory support were moderate to severe dyspnoea, respiratory rate > 30 bpm, or PaO2/FiO2 < 200 mmHg, screened either at hospital admission or ward admission. The decision regarding the choice of treatment was taken by the pulmonologist in charge of the patient’s care, with HFNC usually as the first step after the failure of conventional oxygen therapy^8^, and taking into account the availability of NIRS devices at each centre.

In the HFNC group, heated and humidified oxygen was applied through nasal prongs, at an initial flow rate of 50–60 lpm if tolerated. CPAP was initially set at 8–10 cm H_2_O and then adjusted according to tolerance and clinical response. In the NIV group, a pressure support ventilator mode was adjusted; a high positive end-expiratory pressure (PEEP) and a low support pressure were used to set a tidal volume < 9 ml/kg of predicted body weight^8^. NIRS treatments were applied continuously for at least 48 h while controlling oxygen delivery to obtain a target oxygen saturation measured by pulse oximetry (SpO_2_) of 92–96%^21^. In the NIV and CPAP groups, if the treatment was not tolerated continuously, a minimal duration of 8 h per day, predominantly during the night, was attempted, reaching a mean usage of 22 (4) h/day in NIV and 21 (4) h/day in CPAP (min-P25-median-P75-max 8-22-24-24-24 in both groups). HFNC was not used during breaks in the NIV or CPAP groups due to the limited availability of devices in the first wave of the pandemics. In order to minimize the risks of infection to staff, we applied NIV and CPAP treatments through oronasal or total face non-vented masks attached to single-limb circuits with intentional leak, and placing a low-pressure viral filter preventing exhaled droplet dispersion; in HFNC-treated patients, a surgical mask was put over the nasal prongs^8,9^.

Parallel to the start of NIRS, the ceiling of care was determined considering the patient’s wishes (or those of their representatives), underlying comorbidities, and frailty^22^. A do-not-intubate order was established at the discretion of the attending physician, after discussion with the critical care physician. In case of doubt, the final decision was discussed by the ethical committee at each centre. Intubation was performed when clinically indicated based on the judgment of the responsible physician.

In addition to NIRS treatment, conscious pronation was performed in some patients. Patients were treated and monitored continuously in adapted respiratory wards, with improved monitoring and increased nurse-patient ratio (1:4 to 1:6 in wards, and from 1:2 to 1:4 in high-dependency units).

### Patients’ characteristics

Study data were collected and managed using REDCap electronic data capture tools hosted at ISGlobal (Institut de Salut Global, Barcelona)^23^. We obtained patients’ data from electronic medical records using a modified version of the standardized International Severe Acute Respiratory and Emerging Infection Consortium (ISARIC) COVID-19 case report forms^24^, including: (i) demographics (age, sex, ethnicity); (ii) smoking status; (iii) chronic conditions (cardiac disease, respiratory disease, kidney disease, neoplasm, dementia, obesity, neurological conditions, liver disease, diabetes, and a modified Charlson comorbidity index)^25^; (iv) symptoms at admission and physical signs at NIRS initiation (days since the onset of COVID-19 symptoms, temperature, heart rate, systolic and diastolic blood pressure, respiratory rate, and Quick Sequential Organ Failure Assessment (qSOFA) score)^26^; (v) arterial blood gases at NIRS initiation (PaO_2_/F_I_O_2_ ratio calculated for patients with available PaO_2_, and imputed from SpO_2_ for the 33% of patients without PaO_2_)^27^; (vi) laboratory blood parameters at NIRS initiation; (vii) chest X-ray findings (unilateral or bilateral pneumonia); and (viii) treatment received during admission (highest level of care received outside ICU, ICU admission, NIRS as ceiling of treatment, awake prone positioning, and drug treatments).

### Study outcomes

The primary outcome was treatment failure, defined as endotracheal intubation or death within 28 days of NIRS initiation. Secondary outcomes were 28-day mortality, endotracheal intubation at day 28, in-hospital mortality, and duration of hospital stay.

### Statistical analysis

With an expected frequency of 50% for intubation or death in patients with HARF and treated by NIRS^28^, 300 patients were needed in order to detect a significant difference greater than 20% between the types of NIRS evaluated in the present study, with an alpha risk of 0.05 and a statistical power of 80%.

Characteristics of the patients at baseline according to NIRS treatment were described by mean and standard deviation, median and 25th and 75th percentiles (P25 and P75) and by absolute and relative frequencies, and compared using Chi2, Anova and Kruskal Wallis tests. Given the small number of missing information and that missing were considered at random, we conducted a complete case approach.

Kaplan–Meier curves described the crude event-free rate in each NIRS group and were compared by means of the log-rank test. Multivariable Cox proportional-hazards regression models were used to estimate the hazard ratios (HR) for patients treated with NIV and CPAP as compared to HFNC (the reference group), adjusting for age, sex, and variables found to be significantly different between treatments at baseline (hospital, date of admission and sleep apnea). D-dimer levels and respiratory rate at baseline were also significantly associated with treatment, but since they had missing values for 82 and 41 patients respectively, these variables were only included in a sensitivity analysis.

To account for the potential effect modification, analyses were stratified according to hypoxemia severity (moderate-severe: PaO_2_/F_I_O_2_ < 150 mm Hg; mild-moderate: PaO_2_/F_I_O_2_ ≥ 150 mm Hg)^4^. To assess the potential impact of NIRS treatment settings, we compared outcomes within NIRS-group according to: flow in the HFNC group (> 50 vs. ≤ 50 L/min), pressure in the CPAP group (> 10 vs. ≤ 10 cm H_2_O), and PEEP in the NIV group (> 10 vs. ≤ 10 cm H_2_O).

Sensitivity analyses included: (1) repeating models excluding patients who changed their initial NIRS treatment during the course of the hospitalization to another NIRS treatment (*crossover*, n = 44); (2) excluding patients with missing measured PaO_2_/F_I_O_2_ (n = 123); (3) excluding patients receiving NIRS as ceiling of treatment (n = 140); and (4) additionally adjusting models for, one at a time, D-dimer levels, respiratory rate, systemic corticosteroid use and Charlson index.

All analyses were performed using StataCorp. 2019. *Stata Statistical Software: Release 16*. College Station, TX: StataCorp LLC.

### Consent for publication

All authors have approved the submission and provide consent to publish.

## Results

### Patients' characteristics

Among the 367 patients included in the study, 155 were treated with HFNC (42.2%), 133 with CPAP (36.2%), and 79 with NIV (21.5%).

Most patients were male (72%), and the mean age was 67.5 years (SD 11.2). Chronic conditions were frequent (35% of the sample had a Charlson comorbidity index ≥ 2) and did not differ between NIRS treatment groups, except for sleep apnea (more common in the NIV-treated group, Table 1 and Table S1). At the initiation of NIRS, patients had moderate to severe hypoxemia (median PaO_2_/F_I_O_2_ 125.5 mm Hg, P25-P75: 81–174). Clinical severity and laboratory values were well balanced between the groups (Table 2 and Table S2), except for respiratory rate (higher in patients treated with NIV).

**Table 1:** Patients’ baseline characteristics, according to non-invasive respiratory support group.

Characteristics	All (N = 367*)	High-flow oxygen (N = 155)	CPAP (N = 133)	Non-invasive ventilation (N = 79)	*P* value^§^
Age (years), m (sd)	67.5 (11.2)	66.4 (11.6)	68.5 (11.5)	67.9 (9.7)	0.258
Sex: male, n (%)	265 (72.2%)	111 (71.6%)	99 (74.4%)	55 (69.6%)	0.733
**Ethnicity, n (%)**					0.273
Caucasian	312 (88.1%)	138 (90.2%)	105 (85.4%)	69 (88.5%)	
Latin American	29 (8.2%)	11 (7.2%)	10 (8.1%)	8 (10.3%)	
Other	13 (3.7%)	4 (2.6%)	8 (6.5%)	1 (1.3%)	
**Smoker, n (%)**					0.590
Never	219 (59.7%)	87 (56.1%)	85 (63.9%)	47 (59.5%)	
Former	132 (36%)	62 (40%)	41 (30.8%)	29 (36.7%)	
Active	16 (4.4%)	6 (3.9%)	7 (5.3%)	3 (3.8%)	
**Chronic conditions**					
Charlson index^†^, med (P25-P75)	1 (0–2)	1 (0–2)	1 (0–2)	1 (0–2)	0.870
Charlson index^†^, n (%)					
0	142 (38.7%)	63 (40.7%)	50 (37.6%)	29 (36.7%)	0.898
1	97 (26.4%)	36 (23.2%)	39 (29.3%)	22 (27.9%)	
2	55 (15.0%)	25 (16.1%)	21 (15.8%)	9 (11.4%)	
3	44 (12.0%)	18 (11.6%)	14 (10.5%)	12 (15.2%)	
≥ 4	29 (7.9%)	13 (8.4%)	9 (6.8%)	7 (8.9%)	
Chronic cardiac disease, n (%)	85 (23.2%)	33 (21.3%)	33 (24.8%)	19 (24.1%)	0.762
COPD, n (%)	39 (10.6%)	13 (8.4%)	15 (11.3%)	11 (13.9%)	0.410
Asthma, n (%)	22 (6.0%)	10 (6.5%)	7 (5.3%)	5 (6.3%)	0.905
Sleep apnea syndrome, n (%)	40 (10.9%)	9 (5.8%)	18 (13.5%)	13 (16.5%)	0.022
Chronic kidney disease, n (%)	43 (11.7%)	24 (15.5%)	11 (8.3%)	8 (10.1%)	0.146
Malignant neoplasm, n (%)	36 (9.8%)	17 (11%)	13 (9.8%)	6 (7.6%)	0.714
Obesity, n (%)	82 (22.3%)	31 (20%)	28 (21.1%)	23 (29.1%)	0.259
Hypertension, n (%)	209 (56.9%)	81 (52.3%)	82 (61.7%)	46 (58.2%)	0.266
Dyslipidemia, n (%)	163 (44.4%)	67 (43.2%)	58 (43.6%)	38 (48.1%)	0.756
Diabetes without complications, n (%)	85 (23.2%)	31 (20%)	33 (24.8%)	21 (26.6%)	0.451
Diabetes with complications, n (%)	17 (4.6%)	10 (6.5%)	3 (2.3%)	4 (5.1%)	0.235
Chronic neurological disorder, n (%)	27 (7.4%)	11 (7.1%)	7 (5.3%)	9 (11.4%)	0.252
Chronic hematological disease, n (%)	17 (4.6%)	10 (6.5%)	3 (2.3%)	4 (5.1%)	0.235
Rheumatological disorder, n (%)	27 (7.4%)	8 (5.2%)	14 (10.5%)	5 (6.3%)	0.204
**Admission date, n (%)**^**‡**^					0.008
Before 23 March	121 (33.0%)	66 (42.6%)	32 (24.1%)	23 (20.1%)	
23–28 March	125 (34.1%)	42 (27.1%)	50 (37.6%)	33 (41.8%)	
After 28 March	121 (33.0%)	47 (30.3%)	51 (38.4%)	23 (29.1%)	

*Data on ethnicity were missing in 13 cases.

^†^Modified Charlson comorbidity Index^24^.

^‡^Date of admission was categorized in three groups approximating tertiles.

^§^Chi^2^ test or Fisher exact test (when a cell included < 5 observations).

**Table 2:** Patients’ characteristics at the time of initiating non-invasive respiratory support.

	All (N = 367*)	High-flow oxygen (N = 155)	CPAP (N = 133)	Non-invasive ventilation (N = 79)	*P* value
Days to NIRS from symptom onset, med (P25-P75)	11 (8–13)	11 (9–14)	10 (8–13)	10 (8–13)	0.671
Days to NIRS from hospital admission, med (P25-P75)	2 (1–4)	2 (1–4)	3 (1–5)	2 (1–4)	0.272
Heart rate (bpm), m (sd)	90.1 (16.8)	89.0 (16.4)	90.8 (17.4)	91.1 (16.9)	0.593
Systolic blood pressure (mm Hg), m (sd)	127.8 (20.1)	126.8 (19.1)	129.0 (21.2)	127.9 (20.1)	0.655
Diastolic blood pressure (mm Hg), m (sd)	73.0 (12.9)	73.8 (13.0)	73.6 (13.5)	70.3 (11.6)	0.133
Respiratory rate (breaths/min), m (sd)	26.0 (7.4)	25.1 (6.4)	25.6 (7.3)	28.4 (8.7)	0.005
qSOFA ≥ 1, n (%)^†^	246 (74.5%)	104 (74.8%)	83 (70.3%)	59 (80.8%)	0.270
**Arterial blood gas, m (sd)**					
pH	7.45 (0.06)	7.45 (0.06)	7.45 (0.07)	7.44 (0.05)	0.734
PaO_2_ (mm Hg)	75.0 (26.8)	72.9 (24.1)	76.0 (26.6)	76.4 (30.2)	0.661
PaCO_2_ (mm Hg)	34.0 (5.1)	34.2 (5.0)	33.1 (5.3)	34.7 (5.1)	0.134
SpO_2_ (%), med (P25-P75)	93 (90–95.6)	93 (90–95)	94 (91–96)	93 (90–95.1)	0.091
PaO_2_/F_I_O_2_ (mm Hg)^‡^, med(P25-P75)	125.5 (81–174)	126 (81–174)	126 (82–176)	118 (86–174)	0.999
**Hypoxemia severity, PaO**_**2**_**/F**_**I**_**O**_**2**_** (mm Hg), n (%)**					0.759
≥ 150	126 (35.0%)	51 (32.9%)	47 (37.0%)	28 (35.9%)	
< 150	234 (65.0%)	104 (67.1%)	80 (63.0%)	50 (64.1%)	
**Laboratory values**					
Hemoglobin, g/dL, m (sd)	13.0 (2.1)	13.2 (1.9)	12.7 (2.5)	13.2 (1.7)	0.128
Lymphocyte count, 10^9^/L, GM (sd)	0.79 (1.78)	0.78 (1.89)	0.85 (1.65)	0.72 (1.75)	0.120
Creatinine, mg/dL GM (sd)	0.99 (1.82)	0.97 (1.82)	1.01 (1.76)	0.99 (1.91)	0.856
C-reactive protein, mg/L GM (sd)	50.77 (4.76)	56.81 (4.64)	49.37 (4.21)	43.03 (5.92)	0.460
D-Dimer, ngr/mL, GM (sd)	702 (2.9)	515.2 (2.6)	897.1 (2.8)	785.7 (3.1)	< 0.001
Interleukin-6, pg/mL, GM (sd)	103.4 (3.3)	111.3 (3)	93.6 (3.8)	102.1 (3.3)	0.722
Ferritin, ngr/mL, GM (sd)	1056.5 (2.5)	1066.5 (2.1)	1018.4 (2.7)	1104.6 (2.8)	0.879
**Chest X-ray, n (%)**					0.459
Unilateral pneumonia	15 (4.1%)	4 (2.6%)	7 (5.3%)	4 (5.1%)	
Bilateral pneumonia	352 (95.9%)	151 (97.4%)	126 (94.7%)	75 (94.9%)	

P-value from Chi^2^ test (categorical), Anova (continuous).

*FiO*_*2*_ fraction of inspired oxygen; *GM* Geometric Mean, *NIRS* non-invasive respiratory support, *PaO*_*2*_ arterial partial pressure of oxygen, *PaCO*_*2*_ arterial partial pressure of carbon dioxide, *SpO*_*2*_ oxygen saturation by pulse oximetry.

*Some variables had missing values: 15 in heart rate, 21 in systolic blood pressure, 32 in diastolic blood pressure, 41 in respiratory rate, 102 in arterial pH, 118 in PaO2, 102 in PaCO2, 3 in SpO2, 7 in PaO_2_/F_I_O_2_, 37 in qSOFA, 2 in hemoglobin, 6 in lymphocyte count, 4 in creatinine, 47 in C-reactive protein, 82 in D-Dimer, 213 in interleukin-6, and 151 in ferritin.

^†^Quick Sequential Organ Failure Assessment score ranging from 0 to 3, calculated by adding 1 point for Respiratory rate >  = 22/min, 1 point for change in mental status (doctor diagnosed “altered mental status/confusion”), and 1 point for systolic blood pressure <  = 100 mmHg^25^.

^‡^PaO_2_/F_I_O_2_ was calculated as (PaO_2_ (in mmHg)/F_I_O_2_ (in %)) for 244 patients with available PaO_2_, and estimated using Brown et al.’s formula for patients without PaO_2_^26^. The correlation between measured and calculated PaO_2_/F_I_O_2_ for the 244 patients with complete information was 0.81.

### Treatments

The NIRS treatments applied were not equally distributed among participating hospitals, although HFNC or CPAP were the first NIRS treatment choice at all centers (Table S1). Differences were also found in the NIRS treatments applied according to the date of admission: HFNC was the most frequent treatment early in the period (before 23 March), while CPAP was the most frequent choice in the second and the third periods (Table 1, p = 0.008). Noninvasive respiratory support treatments were applied as ceiling of treatment in 140 patients (38%) (Table 3). Table S3 shows the NIRS settings.

**Table 3:** Inpatient characteristics and treatments according to non-invasive respiratory support group.

	All (N = 367*)	High-flow oxygen (N = 155)	CPAP (N = 133)	Non-invasive ventilation (N = 79)	*P* value
**Highest level of care received outside ICU, n (%)**					< 0.001
Medical ward	204 (55.6%)	75 (48.4%)	104 (78.2%)	25 (31.6%)	
High dependency unit	163 (44.4%)	80 (51.6%)	29 (21.8%)	54 (68.4%)	
ICU admission, n (%)	93 (25.0%)	48 (31.0%)	21 (15.9%)	24 (30.4%)	0.007
NIRS as ceiling of treatment, n (%)	140 (38.1%)	56 (36.1%)	51 (38.3%)	33 (41.8%)	0.701
Awake prone positioning, n (%)	110 (30.1%)	42 (27.1%)	42 (31.6%)	26 (33.3%)	0.457
**Inpatient drug treatment**					
Systemic corticosteroids^†^, n (%)	251 (68.6%)	99 (63.9%)	95 (71.4%)	57 (73.1%)	0.243
Hydroxychloroquine, n (%)	320 (87.2%)	136 (87.7%)	117 (88%)	67 (84.8%)	0.773
Tocilizumab, n (%)	172 (46.9%)	73 (47.1%)	67 (50.4%)	32 (40.5%)	0.378
Lopinavir/ritonavir, n (%)	208 (56.7%)	89 (57.4%)	58 (43.6%)	61 (77.2%)	< 0.001
Azithromycin, n (%)	237 (74.4%)	93 (60.0%)	120 (90.2%)	60 (76.0%)	< 0.001
Anticoagulation, n (%)					0.028
Prophylaxis	210 (57.4%)	104 (67.1%)	65 (48.9%)	41 (52.6%)	
Full treatment	102 (27.9%)	34 (21.9%)	45 (33.8%)	23 (29.5%)	

P-value from Chi^2^ test.

*Anticoagulation had 1 missing value.

^†^Systemic corticosteroids included prednisone (n = 6), methylprednisolone (n = 223), dexamethasone (n = 21) and hydrocortisone (n = 1).

### Primary and secondary outcomes

**Figure 2:**
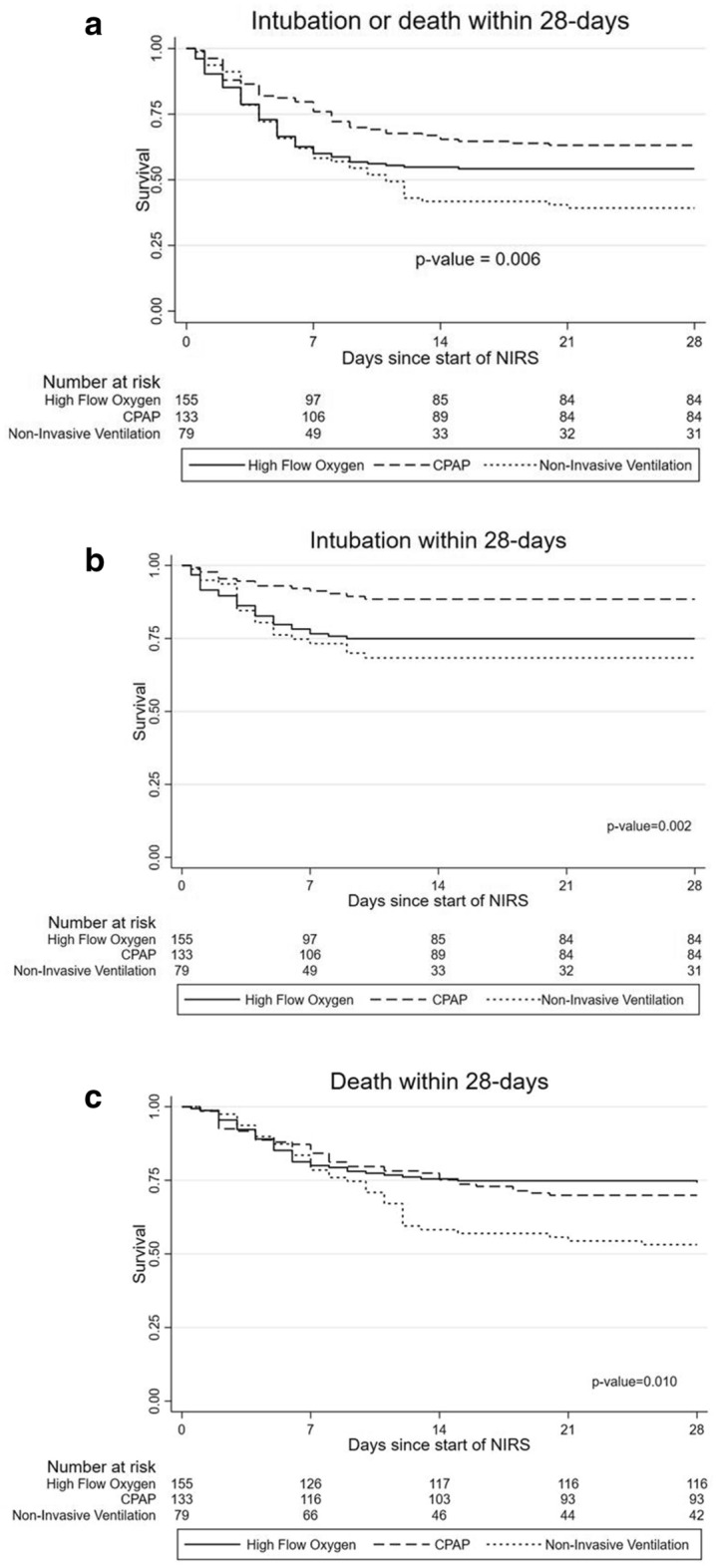
The 28-days Kaplan Meier curves from: (**a**) day starting NIRS to death or intubation; (**b**) day starting NIRS to intubation; and (**c**) day starting NIRS to death. *NIRS* non-invasive respiratory support.

The cumulative percentage of patients who had received intubation or who had died by day 28 (primary outcome) was 45.8% in the HFNC group, 36.8% in the CPAP group, and 60.8% in the NIV group (Fig. 2a). After adjustment, and taking patients treated with HFNC as reference, patients who underwent NIV had a higher risk of intubation or death at 28 days (HR 2.01, 95% CI 1.32–3.08), while those treated with CPAP did not present differences (HR 0.97, 95% CI 0.63–1.50) (Table 4).

**Table 4:** Outcomes by non-invasive respiratory support group.

Outcomes	N = 367	High-flow oxygen (N = 155)	CPAP (N = 133)	Non-invasive ventilation (N = 79)
**Main outcome**				
Death or intubation at day 28 after initiating NIRS	n (%), 168 (45.8%) HR (95% CI) *P* value	71 (45.8%) 1.00	49 (36.8%) 0.97 (0.63–1.50) *P* = 0.891	48 (60.8%) 2.01 (1.32–3.08) *P* = 0.001
**Secondary outcomes**				
Endotracheal intubation during 28 days within NIRS	n (%)^†^, 73 (19.9%) HR (95% CI) *P* value	36 (23.2%) 1.00	14 (10.5%) 0.64 (0.31–1.30) *P* = 0.212	23 (29.1%) 2.38 (1.29–4.39) *P* = 0.006
28-day mortality after initiating NIRS	n (%), 117 (31.9%) HR (95% CI) *P* value	40 (25.8%) 1.00	40 (30.1%) 1.11 (0.65–1.90) *P* = 0.704	37 (46.8%) 2.78 (1.61–4.78) *P* < 0.001
In-hospital mortality*	n (%), 123 (33.5%) HR (95% CI) *P* value	43 (27.7%) 1.00	43 (32.3%) 1.06 (0.63–1.78) *P* = 0.834	37 (46.8%) 2.30 (1.35–3.92) *P* = 0.002
Length of hospital stay^†^	median (P25-P75), 16 (10–25) exp(β) (95% CI)^‡^ *P* value	16 (10–26) 1.00	16 (11–22) 0.95 (0.78–1.15) *P* = 0.598	16 (9–23) 0.89 (0.73–1.10) *P* = 0.284

*CI* confidence interval.

HR Hazard ratio from multivariable survival model adjusted by age, sex, hospital, admission date (tertiles) and sleep apnea. P-value: Wald test.

*In-hospital mortality: at any time during hospital stay, even if > 28 days after initiating NIRS.

^†^Length of hospital stay: admission to discharge-or in-hospital death.

^‡^exp(β): coefficient (exponentiated) from linear regression for Length of hospital stay (log-transformed) adjusted for the same variables as other models. exp(β) can be interpreted as % change in the geometric mean length hospital stay.

As for secondary outcomes, patients treated with NIV had a significantly higher risk of endotracheal intubation, 28-day mortality, and in-hospital mortality than patients treated with HFNC, while no differences were observed between CPAP and HFNC (Fig. 2b,c, Table 4). A total of 73 patients (20%) were intubated during the hospitalization. Among them, 22 (30%) died within 28 days (5/36 in HFNC (14%), 5/14 in CPAP (36%), and 12/23 in NIV (52%) groups, p = 0.007). The patients who had died by day 28 were 117 (31.9%), 91 (65%) of those patients were treated with NIRS as ceiling of treatment and 26 (11.5%) were treated with NIRS not regarded as ceiling of treatment. Days between NIRS initiation and intubation (median (P25-P75) 3 (1–5), 3.5 (2–7), and 3 (3–5), for HFNC, CPAP, and NIV groups respectively; p = 0.341) and the length of hospital stay did not differ between groups (Table 4). Outcomes by hospital are listed in Table S4.

### Stratified and sensitivity analyses

In patients with mild-moderate hypoxaemia, CPAP, but not NIV, treatment was associated with reduced outcome risk compared to HFNC (Table S5). The analyses excluding patients with missing PaO_2_/F_I_O_2_ or receiving NIRS as ceiling of treatment showed similar associations to those observed in the main analysis (Tables S6 and S7, respectively). No differences were found when we performed within NIRS-group comparisons according to settings applied (Table S8).

During the follow-up period, 44 patients (12%) switched to another NIRS treatment: eight (5%) in the HFNC group (treated subsequently with NIV), 28 (21%) in the CPAP group (13 switched to HFNC, and 15 to NIV), and eight (10%) in the NIV group (seven treated with HFNC, and one with CPAP). Excluding these patients showed no relevant changes in the associations observed (Table S9). Additional adjustment for D-dimer, respiratory rate, Charlson index, or treatment with systemic corticosteroids produced very similar results (Table S10).

## Discussion

This study shows that noninvasive ventilation initiated outside the ICU for the treatment of hypoxemic acute respiratory failure secondary to COVID-19 resulted in higher mortality or intubation rate at 28 days (i.e., treatment failure) than high-flow oxygen or CPAP. These results were robust to a number of stratified and sensitivity analyses.

Most previous data on the effectiveness of NIRS treatments in severe COVID-19 patients came from studies which had limited sample sizes and were not designed to compare the different techniques^13–15,17,18^. In the only available study (also observational) comparing NIV, HFNC and CPAP outside the ICU^16^, conducted in Italy, the authors did not find differences between treatments in mortality or intubation at 30 days. The discrepancy between these results and ours may be due to differences in the characteristics of the patients included. First, in the Italian study, the mean PaO_2_/F_I_O_2_ ratio was 152 mm Hg, suggesting a less severe respiratory failure than in our patients (125 mm Hg). In the stratified analysis of our cohort, planned a priori, patients with a PaO_2_/F_I_O_2_ ratio above 150 responded similarly to HFNC and NIV treatments, suggesting that the severity of the hypoxemia might predict the success of NIV, as previously reported in non-COVID patients^4,28,29^. Second, the Italian study did not provide data on PaCO_2_, meaning that the improvements with NIV might have been attributable to the inclusion of some patients with hypercapnic respiratory failure, who were excluded in our study.

Recently, the effectiveness of CPAP or HFNC compared with conventional oxygen therapy was assessed in the RECOVERY-RS multicentric randomized clinical trial, in 1,273 COVID-19 patients with HARF who were deemed suitable for tracheal intubation if treatment escalation was required^20^. In this study, the requirement of intubation or mortality within 30 days (primary outcome) was significantly lower with CPAP (36%) than with conventional oxygen therapy (45%; absolute difference, − 8% [95% CI, − 15% to − 1%], p = 0.03). This improvement was mostly driven by a reduction in the need of intubation, but no differences in mortality were seen (16.7% vs 19.2%, respectively). No significant differences in the main outcome were found between HFNC (44%) vs conventional oxygen therapy (45%; absolute difference, − 1% [95% CI, − 8% to 6%], p = 0.83). The main difference in respect to our study was the better outcomes of CPAP compared with HFNC. However, the RECOVERY-RS study may have been underpowered for the comparison of HFNC vs conventional oxygen therapy due to early study termination and the number of crossovers among groups (11.5% of HFNC and 23.6% of conventional oxygen treated patients).

There are several possible explanations for the poor outcome of COVID-19 patients undergoing NIV in our study. First, NIV has been reported to produce overdistension, compounded by the respiratory effort itself^30^, which could result in ventilation-induced lung injury due to the excessive increases in tidal volumes^28,31^. This risk would be avoided in CPAP and HFNC because they improve oxygenation without changing tidal volume^32,33^. Unfortunately, tidal volume measurements during NIV were not available in our study to support or reject this hypothesis. Second, patient-ventilator asynchronies might have arisen in NIV-treated patients making more difficult their management outside the ICU setting and thereby explaining, at least partially, their worse outcomes. Third, a bench study has recently reported that some approaches to minimize aerosol dispersion can modify ventilator performance^34^. In short, the addition of intentional leaks, as in our study, led to a lower maximal pressure without a significant impact on the work of breathing and without increasing patient-ventilator asynchronies^34^. Then, in the present work, we believe that the availability of trained pulmonologists to adjust ventilator settings may have overcome this aspect. Fourth, non-responders to NIV could have suffered a delay in intubation, but in our study the time to intubation was similar in the three NIRS groups, thus making this explanation less likely. Fifth, we cannot exclude the possibility that NIV implied a more complicated clinical course than HFNC or CPAP. Patients undergoing NIV may require some degree of sedation to tolerate the technique, but unfortunately we have no data on this regard. Furthermore, NIV and CPAP may impair expectoration which could contribute to bacterial infections, although this hypothesis remains unknown with the present data. Finally, we cannot rule out the possibility that NIV was tolerated worse than HFNC or CPAP, which would have reduced adherence and lowered the effectiveness of the therapy. However, the number of patients abandoning their original treatment was nearly twice as high in the CPAP group than in the NIV group.

In the treatment of HARF with CPAP or NIV the interface via which these treatments are applied should be considered, since better outcomes have been reported with a helmet interface than with face masks in non-COVID patients^6,35^ , possibly due to a greater tolerance of the helmet and a more effective delivery of PEEP^36^. As noted above, a single randomized study has evaluated helmet NIV against HFNC in COVID-19^19^, and, in spite of the lower intubation rate in the helmet NIV group, no differences in 28-day mortality were registered. More studies are needed to define the place of treatment with helmet CPAP or NIV in respiratory failure due to COVID-19, together with other NIRS strategies. In our study, CPAP and NIV treatments were applied via oronasal and full face masks, reflecting the fact that most hospitals in our country have little experience with the helmet interface.

Our study supports several guidelines^37,38^ that favor HFNC and CPAP over NIV for the treatment of HARF in COVID-19 patients, but to our knowledge no previous data have been published in support of this recommendation. Furthermore, our results suggest that the severity of the hypoxemic respiratory failure might help physicians to decide which specific NIRS technique could be better for a patient. However, the retrospective design of our study does not allow establishing a causative link between NIV and the worse clinical outcomes observed. Obviously, reaching a definitive conclusion on this point will require further studies with better phenotypic characterization of patients, and considering additional factors implicated in the response to therapies such as the interface used or the monitoring of the inspiratory effort.

This study has some limitations. First, the observational design could have resulted in residual confounding by selection bias. However, the inclusion of patients was consecutive and the collection of variables was really comprehensive. Moreover, NIRS treatment groups exhibited only minor differences which were accounted for in the multivariable and sensitivity analyses thus minimizing the selection bias risk. Although treatment received and outcomes differed by hospital, this fact was taken into account through adjustment. Second, we must be cautious before extrapolating our results to other nonemergency situations. Our study was carried out during the first wave of the pandemics when the healthcare system was overwhelmed and many patients were treated outside ICU facilities. As mentioned above, NIV might have better outcomes in a more controlled setting allowing an optimal critical care^39^. However, the scarcity of critical care resources has remained along the different pandemic surges until now and this scenario is unfortunately frequent in other health care systems around the world. Thus, we believe that our results may be useful for a great number of physicians treating COVID-19 patients around the world. Third, crossovers could have been responsible for differences observed between NIRS treatments but their proportion was small (12%) and our results did not change when these patients were excluded. Fourth, it could be argued that changes in treatment strategies over the timeframe of the study may have led to differential effects of the NIRS. Nevertheless, we do not think it may have influenced our results, because analyses were adjusted for relevant treatments such as systemic corticosteroids^40^ and included the time period as a covariate. And finally, due to the shortage of critical care ventilators at the height of the pandemic, some patients were treated with home devices with limited FiO2 delivery capability and, therefore, could have been undertreated^41,42^. However, as more home devices were used in the CPAP group (81.6% vs. 38% in the NIV group; Table S3), and better outcomes were recorded in the CPAP-treated patients, our result do not support this concern.

The main strength of this study is, in our opinion, its real-life design that allows obtaining the effectiveness of these techniques in the clinical setting. In the current situation with few available data from randomized control trials regarding the best choice to treat COVID-19 patients with noninvasive respiratory support, data from real-life studies like ours may be appropriate^43^. These data are complementary and still useful later on by including some patients usually excluded from randomized studies; patients with do-not-intubate orders are an example and, obviously, they represent a challenge for the physician responsible to decide the best therapeutic strategy.

## Conclusions

In conclusion, the present real-life study shows that, in the context of the pandemic and outside the intensive care unit setting, noninvasive ventilation for the treatment of hypoxemic acute respiratory failure secondary to COVID-19 resulted in higher treatment failure than high-flow oxygen or CPAP. These findings may be relevant for many physicians elsewhere since the successive pandemic surges result in overwhelmed health care systems, leading to the need for severe COVID-19 patients to be treated out of critical care settings.

## Supplementary Information

Supplementary Information.

## Data Availability

All data generated or analyzed during this study are included in this published article and its supplementary information files.

## Abbreviations

CI: Confidence interval
FiO2: Fraction of inspired oxygen
GM: Geometric mean
HARF: Hypoxemic acute respiratory failure
HFNC: High-flow oxygen administered via nasal cannula
HR: Hazard ratio
ICU: Intensive care unit
NIRS: Noninvasive respiratory support
NIV: Noninvasive ventilation
PaO_2_: Arterial partial pressure of oxygen
PaCO_2_: Arterial partial pressure of carbon dioxide
PEEP: Positive end-expiratory pressure
SpO_2_: Oxygen saturation by pulse oximetry
qSOFA: Quick sequential organ failure assessment
