# Supplementary material for: Higher mortality and intubation rate in COVID-19 patients treated with noninvasive ventilation compared with high-flow oxygen or CPAP
Source: Sci Rep. 2022 Apr 20;12:6527. doi: 10.1038/s41598-022-10475-7 (PMC9020755; doi:10.1038/s41598-022-10475-7)
Supplement: Supplementary file 1 — Supplementary Information. [file 41598_2022_10475_MOESM1_ESM.docx]

**SUPPLEMENTAL MATERIAL**

**Higher mortality and intubation rate in COVID-19 patients treated with noninvasive ventilation compared with high-flow oxygen or CPAP**

| **Characteristics** | **All**  **(N =367)** | **High-Flow Oxygen**  **(N =155)** | **CPAP**  **(N = 133)** | **Non-invasive Ventilation**  **(N = 79)** | **P value*** |
| --- | --- | --- | --- | --- | --- |
| **Idiopathic pulmonary fibrosis, n (%)** | 2 (0.5%) | 0 (0%) | 1 (0.7%) | 1 (1.3%) | 0.429 |
| **Other interstitial lung disease, n (%)** | 9 (2.4%) | 7 (4.5%) | 1 (0.7%) | 1 (1.3%) | 0.087 |
| **Liver disease, n (%) No** | 342 (93.2%) | 145 (93.6%) | 126 (94.7%) | 71 (89.9%) | 0.142 |
| Mild | 18 (4.9%) | 7 (4.5%) | 7 (5.3%) | 4 (5.1%) |  |
| Moderate or severe | 7 (1.9%) | 3 (1.9%) | 0 (0%) | 4 (5.1%) |  |
| **Immunocompromised state from solid-organ transplantation, n (%)** | 10 (2.7%) | 4 (2.6%) | 0 (0%) | 6 (7.6%) | 0.004 |
| **Home respiratory therapy** |  |  |  |  |  |
| None, n (%)  Home ventilation, n (%) | 331 (90.2%)  4 (1.1%) | 149 (96.1%)  0 (0%) | 118 (88.7%)  1 (0.8%) | 64 (81.0%)  3 (3.8%) | 0.004  0.016 |
| CPAP, n (%) | 29 (7.4%) | 5 (3.2%) | 14 (10.5%) | 10 (12.7%) | 0.009 |
| LTOT, n (%) | 6 (1.6%) | 1 (0.6%) | 1 (0.8%) | 4 (5.1%) | 0.046 |
| **Platelet count, 10^9^/L, m (sd)** | 219.5 (94.2) | 215.5 (89.1) | 231.0 (93.1) | 207.8 (104.8) | 0.177 |
| **White blood cell count, 10^9^/L, GM (sd)** | 7.3 (1.6) | 7.2 (1.7) | 7.4 (1.5) | 7.4 (1.8) | 0.876 |
| **Neutrophil count, 10^9^/L, GM (sd)** | 5.7 (1.8) | 5.5 (1.8) | 5.8 (1.7) | 6 (1.8) | 0.516 |
| **Glucose, mg/dL, GM (sd)** | 132.3 (1.4) | 130.8 (1.4) | 136.6 (1.4) | 128.5 (1.4) | 0.488 |
| **Hospital , n(%)** |  |  |  |  | <0.001 |
| 1 | 26 (7.1%) | 23 (14.8%) | 1 (0.8%) | 2 (2.5%) |  |
| 2 | 17 (4.6%) | 12 (7.7%) | 0 (0%) | 5 (6.3%) |  |
| 3 | 26 (7.1%) | 0 (0%) | 14 (10.5%) | 12 (15.2%) |  |
| 4 | 38 (10.4%) | 16 (10.3%) | 22 (16.5%) | 0 (0%) |  |
| 5 | 30 (8.2%) | 2 (1.3%) | 21 (15.8%) | 7 (8.9%) |  |
| 6 | 49 (13.4%) | 24 (15.5%) | 16 (12%) | 9 (11.4%) |  |
| 7 | 48 (13.1%) | 21 (13.5%) | 6 (4.5%) | 21 (26.6%) |  |
| 8 | 9 (2.5%) | 0 (0%) | 6 (4.5%) | 3 (3.8%) |  |
| 9 | 43 (11.7%) | 3 (1.9%) | 30 (22.6%) | 10 (12.7%) |  |
| 10 | 81 (22.1%) | 54 (34.8%) | 17 (12.8%) | 10 (12.7%) |  |

##

## **Table S1.** Additional Characteristics of the Patients at Baseline, according to NIRS Group. *Chi^2^ test or Fisher exact test (when a cell included <5 observations) and Anova (continuous). Note: values for some variables were missing: glucose: n=68, white blood cell count n=1, Platelet count n=3, Neutrophil count n=5. *CPAP* continuous positive airway pressure, *GM* Geometric Mean, *LTOT* long-term oxygen therap, *NIRS* non-invasive respiratory support.

| **Symptoms / Signs** | **All**  **(N =367)** | **High-Flow Oxygen**  **(N =155)** | **CPAP**  **(N = 133)** | **Non-invasive Ventilation**  **(N = 79)** | ***P* value** |
| --- | --- | --- | --- | --- | --- |
| **Days to hospital admission from symptom onset, med (P25-P75)** | 7 (5-9) | 7 (5-10) | 7 (5-8) | 7 (5-8) | 0.773 |
| **Temperature (ºC), m(sd)**  **Fever (tem>37.5 at admission or reported previous to admission), n (%)** | 37.2 (1.0)  319 (87.9%) | 37.2 (1.1)  134 (86.5%) | 37.2 (1.0)  120 (90.9%) | 37.2 (1.1)  65 (85.5%) | 0.790  0.401 |
| **Shortness of breath, n (%)** | 235 (64.4%) | 97 (63.4%) | 81 (60.9%) | 57 (72.2%) | 0.241 |
| **Cough, n (%)** | 254 (70.2%) | 109 (71.7%) | 93 (69.9%) | 52 (67.5%) | 0.806 |
| **Confusion, n (%)** | 24 (6.7%) | 7 (4.6%) | 13 (9.8%) | 4 (5.4%) | 0.191 |
| **Fatigue, n (%)** | 209 (60.1%) | 95 (63.8%) | 74 (57.4%) | 40 (57.1%) | 0.475 |
| **Headache, n (%)** | 28 (8.5%) | 8 (5.6%) | 10 (8%) | 10 (15.9%) | 0.051 |
| **Abdominal symptoms*, n (%)** | 102 (29.5%) | 36 (24.5%) | 45 (34.1%) | 21 (31.3%) | 0.200 |
| **Muscle ache, n (%)** | 30 (8.6%) | 15 (9.9%) | 12 (9.2%) | 3 (4.5%) | 0.401 |
| **Chest pain, n (%)** | 93 (27.7%) | 34 (23.6%) | 37 (28.9%) | 22 (34.4%) | 0.257 |
| **Sore throat, n (%)** | 16 (4.4%) | 8 (5.2%) | 6 (4.5%) | 2 (2.5%) | 0.731 |
| **Joint pain, n (%)** | 71 (21.2%) | 25 (17.5%) | 32 (25%) | 14 (21.9%) | 0.315 |

##

## **Table S2**. Presenting symptoms and signs of the patients, according to NIRS group. P-value from Chi^2^ test (categorical), Anova or Kruskal-Wallis test (continuous). *Abdominal pain, vomiting/nausea or diarrhoea. Some variables had missing values: 20 in temperature, 4 in fever at admission, 2 in Shortness of Breath, 5 in cough, 10 in confusion, 19 in fatigue, 37 in headache, 21 in abdominal symptoms, 18 in muscle ache, 31 in chest pain, 39 in Sore throat, 32 in joint pain. *CPAP* continuous positive airway pressure, *NIRS* non-invasive respiratory support.

| **High-flow oxygen (n = 155)*** |  |
| --- | --- |
| Flow, L/min | 60 (50-60) |
| Days of treatment | 5 (2-7) |
| **CPAP (n = 133)** † |  |
| CPAP pressure, cm H_2_O | 12 (10-13) |
| Mask,  Full face  Oronasal | 33 (25%)  100 (75%) |
| Days of treatment | 5 (2-8) |
| **Non-invasive ventilation (n = 79)**‡ |  |
| IPAP, cm H_2_O | 16 (15-18) |
| PEEP, cm H_2_O | 12 (10-12) |
| Mask,  Full face  Oronasal | 17 (21%)  64 (79%) |
| Days of treatment | 5 (3-9) |

## **Table S3.** Non-invasive respiratory support characteristics. Results are expressed as: median (percentiles 25^th^ and 75^th^ ) or n (%). *AIRVO^TM^ 2 (Fisher & Paykel Healthcare, Auckland, New Zealand) n=142; Optiflow (Fisher & Paykel Healthcare, Auckland, New Zealand) n=4; unknown n=9. F_I_O_2_ used in the high-flow oxigen group was 89% (70-96). †Home CPAP devices 107/131 (81.6%). ‡Home mechanical ventilators 30/79 (38%). *CPAP* continuous positive airway pressure, *IPAP* inspiratory positive airway pressure, *PEEP* positive end-expiratory pressure.

|  |  | **Intubation or 28-day mortality** | **28-day mortality** | **Intubation** |
| --- | --- | --- | --- | --- |
| Hospital | n |  |  |  |
| 1 | 26 | 12 (46%) | 3 (12%) | 12 (46%) |
| 2 | 17 | 5 (29%) | 4 (24%) | 4 (24%) |
| 3 | 26 | 12 (46%) | 12 (46%) | 2 (8%) |
| 4 | 38 | 19 (50%) | 12 (32%) | 11 (29%) |
| 5 | 30 | 4 (13%) | 1 (3%) | 3 (10%) |
| 6 | 49 | 16 (33%) | 7 (14%) | 10 (20%) |
| 7 | 48 | 24 (50%) | 15 (31%) | 12 (25%) |
| 8 | 9 | 2 (22%) | 2 (22%) | 0 (0%) |
| 9 | 43 | 21 (49%) | 19 (44%) | 7 (16%) |
| 10 | 81 | 53 (65%) | 42 (52%) | 12 (15%) |
| Total | 367 | 168 (46%) | 117 (32%) | 73 (20%) |

**Table S4.** Outcomes by hospital.

| **Main outcome: Death or intubation at 28 days** | **High-Flow Oxygen**  **(N = 155)** | **CPAP**  **(N = 133)** | | **Non-invasive Ventilation**  **(N = 79)** | |
| --- | --- | --- | --- | --- | --- |
|  |  | HR (95% CI) | *P* | HR (95% CI) | *P* |
| **Hypoxaemic respiratory failure** |  |  |  |  |  |
| Mild-moderate (n=126) | 1.00 | 0.31 (0.12-0.80) | 0.016 | 1.04 (0.46-2.38) | 0.924 |
| Moderate-severe (n=234) | 1.00 | 1.40 (0.85-2.28) | 0.185 | 2.16 (1.30-3.58) | 0.003 |

## **Table S5**. Stratified analysis according to hypoxaemia severity. HR: Hazard ratio from multivariable survival model adjusted by age, sex, hospital, admission date (tertiles) and sleep apnoea. Mild-moderate hypoxaemic respiratory failure defined as PaO_2_/F_I_O_2_ ≥150 mm Hg. Moderate-severe hypoxaemic respiratory failure defined as PaO_2_/F_I_O_2_ <150 mm Hg. *CPAP* continuous positive airway pressure, *CI* Confidence Interval.

| **Outcomes** | **N = 244** | **High-Flow Oxygen**  **(N = 90)** | **CPAP**  **(N = 85)** | **Non-invasive Ventilation**  **(N = 69)** |
| --- | --- | --- | --- | --- |
| **Main outcome** |  |  |  |  |
| Death or intubation at day 28 after initiating NIRS | n (%), 107 (43.9%)  HR (95% CI)  *P* value | 36 (40.0%)  1.00 | 30 (35.3%)  1.00 (0.57-1.77)  *P*=0.993 | 41 (59.4%)  1.99 (1.20-3.28)  *P=*0.008 |
| **Secondary outcomes** |  |  |  |  |
| Endotracheal intubation during 28 days within NIRS | n (%), 51 (20.9%)  HR (95% CI)  *P* value | 24 (26.7%)  1.00 | 8 (9.4%)  0.58 (0.23-1.46)  *P*=0.249 | 19 (27.5%)  1.74 (0.87-3.49)  *P=*0.116 |
| 28-day mortality after initiating NIRS | n (%), 71 (29.1%)  HR (95% CI)  *P* value | 16 (17.8%)  1.00 | 24 (28.2%)  1.51 (0.71-3.21)  *P*=0.281 | 31 (44.9%)  3.83 (1.88-7.81)  *P<*0.001 |
| In-hospital mortality | n (%), 76 (31.1%)  HR (95% CI)  *P* value | 19 (21.1%)  1.00 | 26 (30.6%)  1.24 (0.61-2.55)  *P*=0.555 | 31 (44.9%)  3.07 (1.54-6.08)  *P=*0.001 |
| Length of hospital stay | median (P25-P75), 16 (10-26)  exp(β) (95% CI)*  *P* value | 17.5 (11-27)  1.00 | 16 (10-26)  1.03 (0.80-1.34)  *P*=0.807 | 15 (9-23)  0.95 (0.74-1.22)  *P*=0.705 |

## **Table S6**. Sensitivity analysis restricted to patients with measured PaO_2_/F_I_O_2_ . HR: Hazard ratio from multivariable survival model adjusted by age, sex, hospital, admission date (tertiles) and sleep apnoea. *exp(β): coefficient (exponentiated) from linear regression for Length of hospital stay (log-transformed) adjusted for the same variables as other models. exp(β) can be interpreted as % change in the GM length hospital stay. P-value: Wald test. In-hospital mortality: at any time during hospital stay, even if >28 days after initiating NIRS. Length of hospital stay: admission to discharge-or in-hospital death. *CI* Confidence Interval, *NIRS* non-invasive respiratory support.

| **Outcomes** | **N = 227** | **High-Flow Oxygen**  **(N = 99)** | **CPAP**  **(N = 82)** | **Non-invasive Ventilation**  **(N = 46)** |
| --- | --- | --- | --- | --- |
| **Main outcome** |  |  |  |  |
| Death or intubation at day 28 after initiating NIRS | n (%), 77 (33.9%)  HR (95% CI)  *P* value | 36 (36.4%)  1.00 | 15 (18.3%)  0.69 (0.34-1.39)  *P*=0.302 | 26 (56.5%)  3.19 (1.72-5.94)  *P<*0.001 |
| **Secondary outcomes** |  |  |  |  |
| Endotracheal intubation during 28 days within NIRS | n (%), 73 (32.2%)  HR (95% CI)  *P* value | 36 (36.4%)  1.00 | 14 (17.1%)  0.66 (0.32-1.35)  *P*=0.252 | 23 (50.0%)  2.76 (1.44-5.26)  *P=*0.002 |
| 28-day mortality after initiating NIRS | n (%), 26 (11.5%)  HR (95% CI)  *P* value | 5 (5.1%)  1.00 | 6 (7.3%)  1.37 (0.41-4.53)  *P*=0.609 | 15 (32.6%)  8.29 (2.97-23.14)  *P<*0.001 |
| In-hospital mortality | n (%), 32 (14.1%)  HR (95% CI)  *P* value | 8 (8.1%)  1.00 | 9 (11.0%)  1.78 (0.61-2.55)  *P*=0.261 | 15 (32.6%)  7.62 (2.88-20.13)  *P<*0.001 |
| Length of hospital stay | median (P25-P75), 18 (14-29)  exp(β) (95% CI)*  p-value | 18 (13-34)  1.00 | 18 (14-26)  0.99 (0.80-1.25)  *p*=0.994 | 16.5 (13-25)  0.89 (0.70-1.12)  *p*=0.319 |

## **Table S7**. Sensitivity analysis excluding patients who received NIRS as ceiling of treatment. HR: Hazard ratio from multivariable cox model adjusted by age, sex, hospital (except 28-day mortality and in-hospital mortality because of model instability), admission date (tertiles) and sleep apnoea. *exp(β): coefficient (exponentiated) from linear regression for Length of hospital stay (log-transformed) adjusted for the same variables as other models. exp(β) can be interpreted as % change in the GM length hospital stay. P-value: Wald test. In-hospital mortality: at any time during hospital stay, even if >28 days after initiating NIRS. Length of hospital stay: admission to discharge-or in-hospital death. *CI* Confidence Interval, *NIRS* non-invasive respiratory support.

|  | **High-Flow Oxygen** | | **CPAP** | | **Non-invasive Ventilation** | |
| --- | --- | --- | --- | --- | --- | --- |
|  | HR (95% CI) | *P* | HR (95% CI) | *P* | HR (95% CI) | *P* |
| **Intubation or death 28 days** |  |  |  |  |  |  |
| Low | 1.00 |  | 1.00 |  | 1.00 |  |
| High | 1.42 (0.74-2.74) | 0.298 | 1.21 (0.59-2.50) | 0.593 | 1.40 (0.58-3.36) | 0.448 |
| **Death at 28-days** |  |  |  |  |  |  |
| Low | 1.00 |  | 1.00 |  | 1.00 |  |
| High | 2.46 (0.69-8.71) | 0.164 | 1.10 (0.48-2.51) | 0.827 | 2.04 (0.75-5.53) | 0.162 |

## **Table S8**. Within NIRS-group of treatment comparisons according to settings applied. HR: Hazard ratio from multivariable survival model adjusted by age, sex, hospital, admission date (tertiles) and sleep apnoea. Low: High-flow oxygen = flow ≤50 L/min; CPAP = pressure ≤10 cm H_2_O; Non-invasive ventilation = Positive end-expiratory pressure ≤10 cm H_2_O. *CI* Confidence Interval, *CPAP* continuous positive airway pressure, *NIRS* non-invasive respiratory support.

| **Outcomes** | **N = 323** | **High-Flow Oxygen**  **(N = 147)** | **CPAP**  **(N = 105)** | **Non-invasive Ventilation**  **(N = 71)** |
| --- | --- | --- | --- | --- |
| **Main outcome** |  |  |  |  |
| Death or intubation at day 28 | n (%), 145 (44.9%)  HR (95% CI)  *P* value | 66 (44.9%)  1.00 | 34 (32.4%)  0.93 (0.57-1.52)  *P*=0.774 | 45 (63.4%)  2.27 (1.45-3.55)  *P<*0.001 |
| **Secondary outcomes** |  |  |  |  |
| Intubation, (28 days within NIRS) | n (%) 67 (20.7%)  HR (95% CI)  *P* value | 33 (22.4%)  1.00 | 11 (10.5%)  0.67 (0.30-1.48)  *P*=0.321 | 23 (32.4%)  2.61 (1.38-4.92)  *P*=0.003 |
| 28-day mortality | n (%) 100 (31.0%)  HR (95% CI)  *P* value | 38 (25.9%)  1.00 | 28 (26.7%)  1.01 (0.56-1.81)  *P*=0.984 | 34 (47.9%)  3.23 (1.82-5.75)  *P<*0.001 |
| In-hospital mortality | n (%) 105 (32.5%)  HR (95% CI)  *P* value | 41 (27.9%)  1.00 | 30 (28.6%)  0.97 (0.55-1.73)  *P*=0.930 | 34 (47.9%)  2.74 (1.56-4.81)  *P*=*<*0.001 |
| Length of hospital stay | Median (P25-P75), 16 (10-23)  exp(β)* (95% CI)  *P* value | 16 (10-26)  1.00 | 16 (10-20)  0.92 (0.77-1.11)  *P*=0.403 | 15 (9-23)  0.91 (0.74-1.11)  *P*=0.356 |

## **Table S9.** Sensitivity analysis restricted to patients who did not change NIRS. HR: Hazard ratio from multivariable survival model adjusted by age, sex, hospital, admission date (tertiles) and sleep apnoea. *exp(β): coefficient (exponentiated) from linear regression for Length of hospital stay (log-transformed) adjusted for the same variables as other models. P-value: Wald test. In-hospital mortality: death during hospital stay (even if >28 days after initiating NIRS). Length of hospital stay: admission to discharge-or in-hospital death. *CI* Confidence Interval, *CPAP* continuous positive airway pressure, *NIRS* non-invasive respiratory support.

| **Main Outcome** |  | **High-Flow Oxygen** | **CPAP** | **Non-invasive Ventilation** |
| --- | --- | --- | --- | --- |
| **Adjusted for:** | n outcome/n subsample (%) |  | HR (95% CI) | HR (95% CI) |
| **Log-D-dimer (ngr/mL)** | 121/285 (42.5%) | 1.00 | 1.20 (0.72-2.01) | 2.22 (1.35-3.67) |
| **Respiratory Rate (breaths/min)** | 148/326 (45.4%) | 1.00 | 1.00 (0.62-1.59) | 1.87 (1.18-2.96) |
| **Charlson Index** | 168/367 (45.8%) | 1.00 | 0.97 (0.63-1.50) | 1.98 (1.30-3.04) |
| **Corticosteroids (yes/no)** | 167/366 (45.6%) | 1.00 | 0.95 (0.61-1.47) | 1.95 (1.27-2.99) |

## **Table S10**. Sensitivity analysis: survival analysis for risk of intubation or death at 28-days with additional covariates adjustment. HR: Hazard ratio from multivariable survival model adjusted for the listed covariate (row) and age, sex, hospital, admission date (tertiles) and sleep apnoea. D-dimer (ngr/mL) was log-transformed. *CI* confidence interval, *CPAP* continuous positive airway pressure.
